# Triangulating Timing, Tropism and Burden of Sarcoma Metastases: Toward Precision Surveillance and Therapy in a Real-World-Time Cohort

**DOI:** 10.3390/cancers17182944

**Published:** 2025-09-09

**Authors:** Philip Heesen, Dario Feusi, Bettina Vogel, Gabriela Studer, Bruno Fuchs, on behalf of the Swiss Sarcoma Network

**Affiliations:** 1 Faculty of Medicine, University of Zurich, Raemistrasse 71, 8006 Zurich, Switzerland; 2 Faculty of Health Sciences & Medicine, University of Lucerne, Frohburgstrasse 3, 6002 Luzern, Switzerland; 3 Swiss Sarcoma Network SSN, LUKS Sarcoma-IPU, University Teaching Hospital LUKS, Spitalstrasse, 6000 Lucerne, Switzerland; 4 Department of Radiation Oncology, LUKS Sarcoma-IPU, University Teaching Hospital LUKS, Spitalstrasse, 6000 Lucerne, Switzerland; 5 Department of Orthopaedics and Trauma, LUKS Sarcoma-IPU, University Teaching Hospital LUKS, Spitalstrasse, 6000 Lucerne, Switzerland; 6 Department of Orthopaedics and Trauma, Kantonsspital Winterthur, KSW Sarcoma Center, Brauerstrasse 15, 8400 Winterthur, Switzerland

**Keywords:** soft-tissue sarcoma, metastasis, oligometastatic disease, real-world-time data, surveillance strategies, organ tropism, precision medicine

## Abstract

**Background**: Sarcoma surveillance guidelines still apply uniform imaging intervals based on tumor grade and stage that ignore histotype-specific metastatic behavior. We prospectively analyzed metastatic timing, organ tropism, and lesion burden across a real-world sarcoma cohort to generate an evidence base for risk-adapted follow-up and treatment stratification. **Methods**: In a prospective multicenter study, 1850 patients with suspected sarcoma were screened. SHAPEHub, a real-world-time data warehouse, captured clinicopathological variables and imaging. Adults with histologically confirmed soft-tissue or bone sarcoma (n = 295) formed the analytic cohort. Metastases were classified as synchronous (≤6 months) or metachronous (>6 months), lung-only versus multi-organ, and oligometastatic (≤5 lesions, ≤2 organs) versus polymetastatic. TTME was illustrated with Kaplan–Meier curves for the full cohort (descriptive); where subgroup comparisons are shown, log-rank tests are reported. **Results**: Ninety-three patients (31.5%) developed metastases after a median follow-up of 20.9 months. Metastatic risk was front-loaded: 36.6% were synchronous, and 67.8% of metachronous events occurred within year 1. The lung was the initial site in 62.4% of events, bone in 18.3%, and liver in 11.8%. Half of the lung-metastatic patients remained pulmonary-confined; the remainder followed a multi-organ route involving bone and lymph nodes. Oligometastatic spread predominated in the lung-only subgroup (61%) versus multi-organ (28%). Histotype influenced both timing and tropism: angiosarcoma and Ewing sarcoma metastasized earliest (median 3.7 and 5.0 months) and multi-organ; leiomyosarcoma and UPS were lung-dominant; Ewing sarcoma and epithelioid haemangioendothelioma were bone-tropic; and angiosarcoma was liver-tropic. **Conclusions**: Metastatic sarcoma displays three intersecting dimensions—early versus late onset, organ-specific tropism, and oligo- versus polymetastatic burden—none of which are addressed by the current “one-size-fits-all” surveillance. Recognizing these patterns delineates windows for tailored imaging and stratified therapy selection (e.g., local ablation for oligometastatic lung disease, intensified systemic regimens for early, polymetastatic spread). These findings lay the groundwork for precision-adapted surveillance and treatment protocols. Pattern-stratified trials and health-economic evaluations are now needed to assess whether this approach improves outcomes and optimizes resource allocation.

## Simple Summary

Patients with sarcoma—the rare cancers of soft tissue and bone—do not all have the same clinical course once the tumor is removed. By following nearly 300 adults in a nationwide, real-time database, we discovered that metastases (new tumors appearing elsewhere) differed when they developed, where they landed, and how many appeared. About one-third of patients already had—or quickly developed—metastases within six months, while others stayed free of spread for many years. Many tumors went only to the lungs and stayed there, but some jumped rapidly to the bones, liver, or lymph nodes. Roughly half the patients had only a few small spots that could be treated with surgery or focused radiation; the rest had many lesions in several organs and needed immediate drug therapy. These patterns suggest that everyone should not get the same scan schedule or the same first-line treatment. Tailoring follow-up and therapy to a tumor’s timing, destination, and tumor-load could improve care while sparing low-risk patients unnecessary tests.

## 1. Introduction

Soft-tissue and bone sarcomas constitute a rare, heterogeneous family of malignancies whose metastatic behavior differs markedly by histologic subtype [1]. Current follow-up recommendations from the European Society for Medical Oncology (ESMO) and the National Comprehensive Cancer Network (NCCN) assign uniform imaging intervals largely on the basis of tumor grade and stage [2,3,4]. This “one-size-fits-all” paradigm risks under-surveillance of aggressive phenotypes, over-surveillance and over-treatment of indolent tumors, and exposes patients to avoidable imaging and therapeutic burden while also inflating healthcare costs.

Metastatic spread in sarcoma is governed by three inter-linked dimensions: temporal dynamics: synchronous (≤6 months) versus metachronous (>6 months) dissemination [5,6]; spatial organ tropism; and metastatic burden ranging from oligometastatic disease (typically ≤3–5 lesions amenable to local control) to polymetastatic dissemination [7,8,9,10,11,12,13,14]. While the lung remains the dominant target [15], several histotypes exhibit characteristic extra-pulmonary patterns: bone involvement in Ewing sarcoma, hepatic spread in angiosarcoma, and nodal metastases in epithelioid sarcoma, among others [16]. In addition, late-metastasizing entities such as clear-cell sarcoma or low-grade fibromyxoid sarcoma frequently evade the conventional five-year surveillance window. Conversely, angiosarcoma and Ewing sarcoma may metastasize within weeks, demanding intensified early imaging [17,18,19]. These divergent timelines are clinically consequential. Early-appearing metastases tend to signal intrinsically aggressive biology that benefits from close surveillance and rapid intervention, whereas late-occurring lesions in indolent histotypes invite a more measured, long-term follow-up strategy. Understanding where a patient’s tumor falls on this temporal spectrum is therefore pivotal for balancing diagnostic yield against surveillance intensity and therapeutic burden. Equally, recognizing metastatic burden and timing has therapeutic consequences: a patient with a handful of lung nodules detected within months of diagnosis differs fundamentally from one who presents years later with multi-organ disease, and who is unlikely to derive identical benefit from the same type of therapy; for instance, chemotherapy. Failing to stratify trials and clinical decisions by these parameters may obscure true treatment effects and exaggerate toxicity–efficacy trade-offs.

Despite these observations, the systematic and prospective evaluation of the temporal, spatial, and burden-related metastatic variation across sarcoma subtypes is scarce. Existing series are often retrospective, single-centered, and underpowered for rare histologies [5,6,12]. Crucially, no large real-world-time data set has yet been leveraged to design a histotype-adapted surveillance algorithm.

The present multicenter study characterizes both the timing, organ distribution, and lesion burden of metastases across a broad sarcoma cohort and aims to inform precision therapy and surveillance. We therefore set out to generate a comprehensive, prospectively-acquired picture of sarcoma dissemination across three complementary axes. First, we quantified the temporal course of metastasis, discriminating between synchronous and metachronous onsets. Second, we mapped the spatial footprint of disease by cataloguing both initial and cumulative organ tropisms. Third, we evaluated metastatic burden, contrasting oligometastatic presentations—typically ≤3–5 lesions amenable to local control—with polymetastatic spread. In addition, we traced sequential progression pathways to elucidate how tumors transition from one compartment to another. By triangulating these dimensions, this study provides a biologic foundation for risk-adapted surveillance and for a more granular stratification of patients in therapeutic trials.

## 2. Materials and Methods

### 2.1. Study Design and Setting

We performed a prospective, multicenter observational cohort study within the Swiss Sarcoma Network (SSN). All consecutive patients evaluated for a suspected sarcoma at participating SSN institutions were entered into SHAPEHub (SHAPE4PM, Schindellegi, Switzerland), a real-world-time data warehouse that prospectively captures clinical, radiological, and pathological variables in a harmonized format.

### 2.2. Eligibility Criteria

Inclusion criteria were: (i) age ≥ 18 years; (ii) histologically-confirmed soft-tissue or bone sarcoma according to the 2020 WHO classification; and (iii) treatment and/or follow-up in an SSN institution between August 2017 and February 2023. Exclusion criteria comprised benign/borderline tumors, Kaposi sarcoma, and insufficient baseline data.

### 2.3. Data Collection and Quality Assurance

Demographic, clinicopathological, and treatment data were prospectively collected with SHAPEHub. Automated integrity checks (range, type, and logic rules) and quarterly manual audits ensured <3% missingness across the core variables. Histological grade followed FNCLCC criteria, and staging employed the AJCC 8th edition. All imaging and pathology reports were centrally reviewed by the SSN weekly tumor board.

### 2.4. Surveillance Imaging Protocol

Baseline work-up included contrast-enhanced thoraco-abdominopelvic computed tomography (CT), or whole-body MRI (WBMRI) for myxoid liposarcoma [15]. Follow-up imaging adhered to the SSN consensus protocols which align with the ESMO/NCCN guidelines [2,3,4] as follows: for high-grade tumors, CT every 3–4 months for years 1–2, then every 6 months until year 5; for low-grade tumors, CT every 6–12 months. WBMRI supplanted CT for histotypes with a predominantly extra-pulmonary spread (e.g., myxoid liposarcoma, epithelioid haemangioendothelioma). During the 2020/2021 COVID-19 surges, SSN centers preserved oncologic imaging capacity, and the above surveillance cadence remained our target schedule. Occasional short operational delays did not alter the protocol.

### 2.5. Definitions and Outcomes

Metastasis was radiologically or histologically confirmed distant disease. Metastatic burden was categorized at first presentation as oligometastatic (≤5 radiologically detectable lesions across a maximum of two organs, each potentially amenable to local control) or polymetastatic (>5 lesions or diffuse multi-organ involvement). Synchronous metastases were diagnosed ≤6 months from primary tumor diagnosis; metachronous metastases occurred >6 months thereafter [5,19]. For descriptive timing analyses, we used time-to-first metastatic event (TTME), defined as the time interval from index histopathologic diagnosis to the first radiologic or histologic confirmation of distant disease. Patients with metastases identified at baseline staging (metastatic at presentation) are displayed as events at *t* = 0; instances where staging preceded histologic sign-out, yielded negative intervals that were normalized to zero. In contrast, metastasis-free survival (MFS) is conventionally defined only in patients without metastasis at diagnosis (M0) and was not used for inferential comparisons in this report.

### 2.6. Statistical Analysis

Continuous variables are presented as medians with interquartile ranges (IQR) and compared using the Mann–Whitney U test. Categorical data are expressed as counts and percentages; comparisons employed χ^2^ or Fisher’s exact tests. The TTME was illustrated with Kaplan–Meier curves for the full cohort (descriptive); where subgroup comparisons are shown, log-rank tests are reported. The statistical significance was set at *p* < 0.05 (two-sided). Analyses were performed in R v4.3.2 (The R Foundation for Statistical Computing).

## 3. Results

### 3.1. Subsection

**Figure 1:**
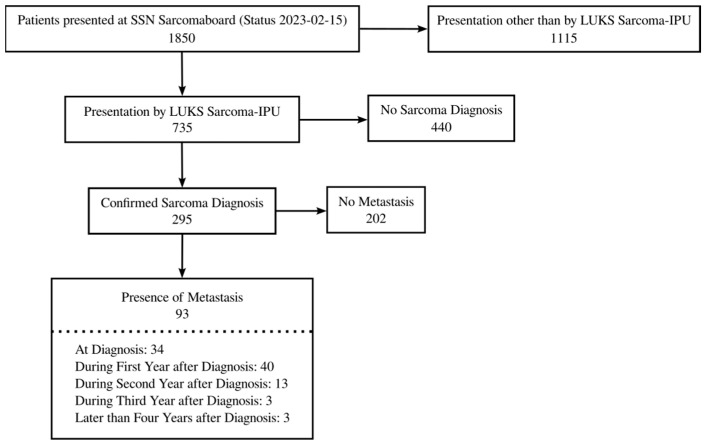
This flowchart provides an overview on the selection process for patients to be included in this study.

Between August 2017 and February 2023, we screened 1850 individuals with suspected sarcoma across the Swiss Sarcoma Network (SSN) centers. After histopathological confirmation, 295 patients (40.1%) were diagnosed with soft-tissue or bone sarcoma and constituted the analytic cohort (Figure 1). Ninety-three patients (31.5%) developed metastatic disease during follow-up, whereas 202 (68.5%) remained metastasis-free.

### 3.2. Baseline Characteristics

Baseline clinicopathological features are summarized in Table 1. Median age at diagnosis was 58.1 years (interquartile range [IQR] 46.7–71.2) with a balanced sex distribution (151/295; 51.2% male). High-grade tumors (FNCLCC G3) were significantly more frequent among metastatic patients (69.9% vs. 37.1%; *p* < 0.001). Median primary-tumor size showed a non-significant trend toward larger lesions in the metastatic group (90 mm vs. 80 mm; *p* = 0.175). Histotypes enriched in the metastatic cohort included angiosarcoma, leiomyosarcoma, and Ewing sarcoma.

### 3.3. Incidence and Timing of Metastasis

**Figure 2:**
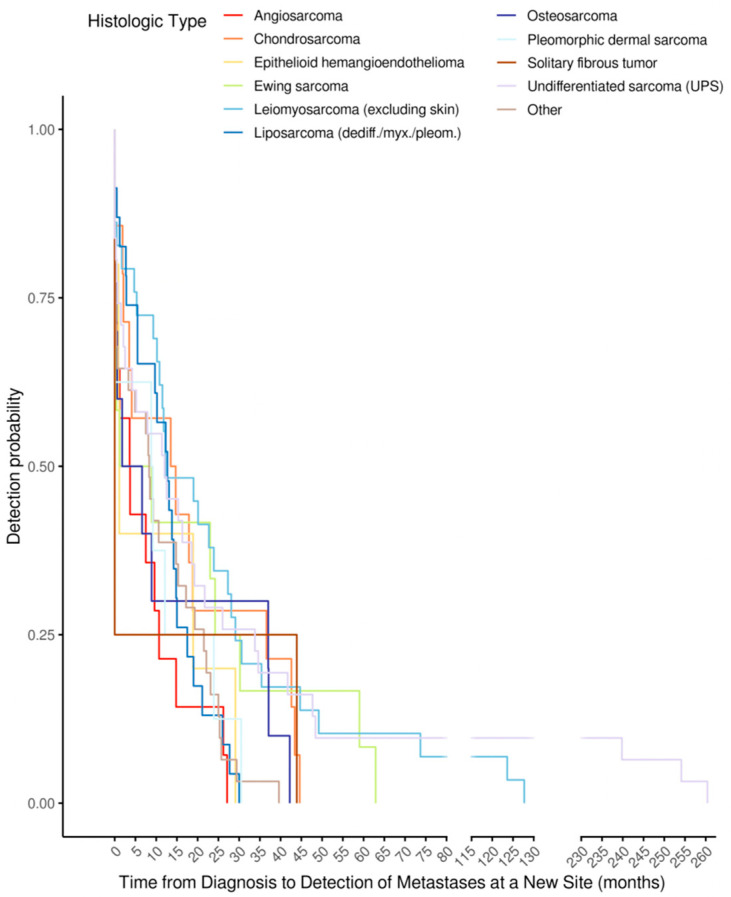
Time-to-first metastatic event (TTME) from index diagnosis for the full cohort. Patients metastatic at presentation are shown at *t* = 0. In cases where baseline staging preceded histopathology sign-out, negative intervals were normalized to zero.

Overall median follow-up was 20.9 months (IQR 9.9–36.2). Metastatic events clustered early: 34/93 patients (36.6%) presented with synchronous metastases (≤6 months from diagnosis), and a further 40/59 metachronous events (67.8%) arose within the first year. The time-to-first metastatic event (TTME) curve shows the steepest drop within the first 12 months. Events at *t* = 0 reflect patients who were metastatic at presentation, as detected on baseline staging (Figure 2).

### 3.4. Anatomic Distribution of First Metastatic Sites

The lung was the predominant first metastatic site (58/93; 62.4%), followed by bone (17/93; 18.3%), lymph nodes (14/93; 15.1%), and liver (11/93; 11.8%) (Table 2a). Subtype-specific organ tropism was evident:

- Leiomyosarcoma and undifferentiated pleomorphic sarcoma (UPS) showed strong pulmonary predilection (78.6% and 72.2%, respectively).
- Ewing sarcoma and epithelioid hemangioendothelioma (EHE) favored bone (83.3% and 66.7%), in line with large Ewing-specific series [20].
- Angiosarcoma and EHE exhibited increased hepatic involvement (14.3% and 66.7%).

### 3.5. Cumulative Metastatic Burden

When all the metastatic events were considered, pulmonary involvement remained the most common (78/93; 83.9%). Secondary spread frequently affected the bone (37/93; 39.8%) and lymph nodes (30/93; 32.3%) (Table 2b).

### 3.6. Metastatic Burden at First Presentation

Details are summarized in Table 3. At the initial metastatic diagnosis, 41/93 patients (44%) displayed an oligometastatic pattern—defined as ≤5 lesions confined to a maximum of two organs—whereas 52/93 (56%) presented with polymetastatic disease. Oligometastatic burden was disproportionately associated with the lung-only trajectory (28/46; 61%) compared with the multi-organ pathway (13/47; 28%). Timing also differed: 14/34 synchronous cases (41%) and 27/59 metachronous cases (46%) were oligometastatic, suggesting that lesion count alone does not fully account for biology; rather, it interacts with timing and organ tropism to define treatment opportunity windows.

### 3.7. Temporal Dynamics Across Histotypes

**Figure 3:**
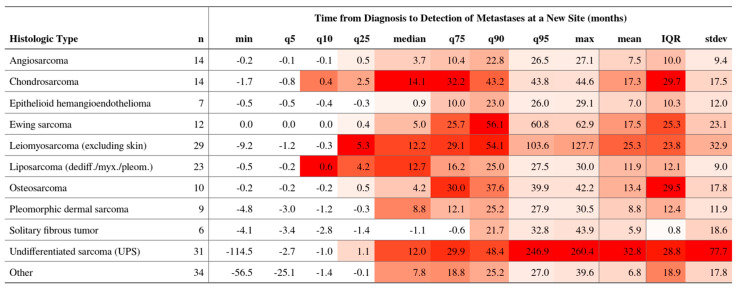
Time from diagnosis to detection of metastases at a new site (months), stratified by histologic subtype (n per row shown). Columns report the minimum, 5th, 10th, and 25th percentiles; median; 75th, 90th, and 95th percentiles; maximum; mean; interquartile range (IQR = q75 − q25); and standard deviation. Negative values indicate cases where the metastatic site was detected prior to or at the time of the index diagnosis due to back-dating or pre-diagnostic imaging.

The median time to first metastasis varied markedly by histotype (Figure 3). Angiosarcoma progressed fastest (median 3.7 months; IQR 0.5–22.8), followed by Ewing sarcoma (5.0 months; IQR 0.4–25.7). Chondrosarcoma (14.1 months; IQR 2.5–32.2) and dedifferentiated liposarcoma (12.7 months; IQR 4.2–16.2) displayed delayed spread.

### 3.8. Metastatic Progression Pathways

**Figure 4:**
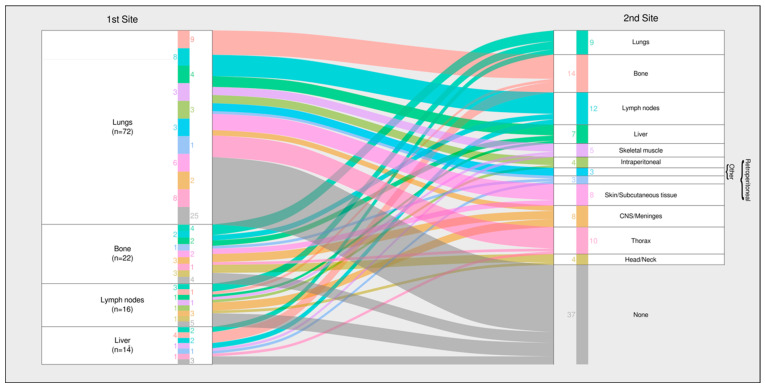
Histotype-specific time-to-first metastatic event (TTME). Values at *t* ≤ 0 indicate metastases identified during baseline staging around the diagnostic work-up and are displayed at *t* = 0.

Sequential mapping (Figure 4) identified two dominant trajectories:

1. Lung-dominant pathway—About half of the patients with an initial pulmonary metastasis remained confined to the lungs, particularly those with leiomyosarcoma, UPS, and osteosarcoma.
2. Multi-organ pathway—The remainder progressed to extrapulmonary sites, most commonly the bone and lymph nodes, a pattern typical of angiosarcoma and Ewing sarcoma.

### 3.9. Key Clinical Signals

**Figure 5:**
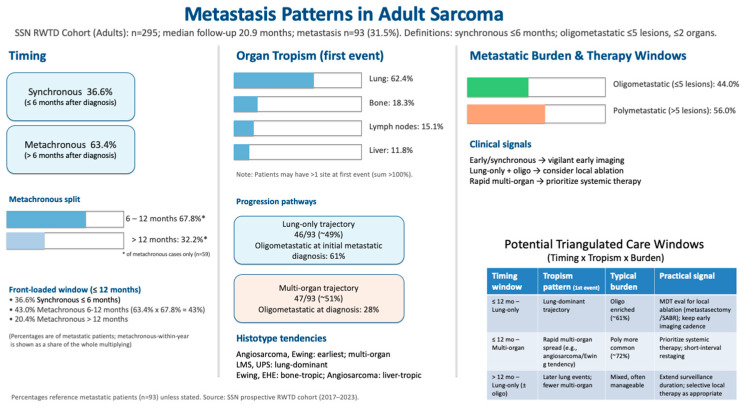
Integrated summary of first-metastasis patterns in adult sarcoma (SSN prospective RWTD cohort).

Early temporal clustering, histotype-specific organ tropism, and burden stratification (oligo-versus polymetastatic) delineate the windows for tailored imaging and burden-informed systemic or local therapy selection. Detailed clinical implications are developed in Section 4 (Discussion). To aid clinical interpretation, Figure 5 integrates timing (synchronous vs. metachronous), organ tropism at the first event, and metastatic burden (oligo vs. poly) into a single visual summary with example care windows.

## 4. Discussion

Our prospective, multicenter analysis reveals three complementary dimensions of sarcoma dissemination. First, metastatic risk is markedly front-loaded with more than one-third of affected patients presenting with synchronous disease (≤6 months), and a further two-thirds of metachronous events emerging within the first year of follow-up. Second, the cohort displays a clear spatial dichotomy. For roughly half of the patients who develop pulmonary disease, the lesion remains confined to the lungs, whereas the remaining patients embark on a multi-organ trajectory that typically incorporates bone and lymph node involvement [21]. Third, metastatic burden diverges sharply, that is, 44% of patients manifest an oligometastatic pattern (≤5 lesions, ≤2 organs) at first presentation, while 56% are polymetastatic. Collectively, these findings underline the inadequacy of the surveillance and treatment frameworks that rely solely on grade and stage. An integrative schematic (Figure 5) summarizes these relationships and illustrates the pragmatic windows for imaging intensity, and local versus systemic therapy selections.

The coexistence of lung-only and multi-organ phenotypes suggests distinct biological programs [22,23,24]. Lung-confined spread may reflect early vascular seeding with limited subsequent adaptability. This pattern is amenable to local interventions, such as metastasectomy, stereotactic ablative radiotherapy, or radiofrequency ablation. By contrast, rapid systemic progression—typical of angiosarcoma and Ewing sarcoma—demands vigilant early imaging and early initiation of systemic or combined-modality therapies. Because our median follow-up was intentionally short and focused on pattern recognition, we did not analyze survival, progression-free survival, or patient-reported outcomes. Linking these dissemination signatures to treatment efficacy and long-term prognosis will require longer, ideally international, follow-up. Even so, recognizing metastatic patterns already delivers practical value because it guides imaging cadence and supplies a biologic stratification framework for future trials (whether evaluating intensified systemic therapy for aggressive polymetastatic disease, or focal ablative approaches in the oligometastatic setting.)

Parallel stratification by synchronous versus metachronous onset refines this picture. Synchronous dissemination signals intrinsically aggressive biology, whereas late events in indolent histotypes (e.g., clear-cell sarcoma) highlight the inadequacy of a fixed five-year follow-up. Moreover, mounting real-world evidence shows that the baseline outcomes for polymetastatic sarcoma have improved—partly due to the wider adoption of focal therapies—causing recent systemic trials [25,26]) to underperform against control arms. Understanding whether a cohort is enriched for oligo- or polymetastatic disease, and synchronous or late-metastatic disease, is therefore critical for both trial design and clinical decision-making. Adequate monitoring and instant access to respective data are therefore critical [27,28,29].

Retrospective single-center series have hinted at organ-specific tropism. Our multicenter data corroborate those findings and add temporal and burden dimensions. Current ESMO and NCCN recommendations remain grade-centered and time-invariant [2,3,4]. Incorporating histotype, timing, organ risk, and burden could rationalize imaging frequency. This could spare indolent tumors from unnecessary radiation while safeguarding early-spreading entities. While skeletal MRI is highly sensitive for osseous metastases, FDG PET–CT or PET–MRI can complement both detection and the treatment-response assessment, particularly for osseous and mediastinal disease in selected histotypes. Our results thus supply the empirical substrate for ongoing guideline refinement.

The patterns identified above delineate a spectrum of surveillance and treatment needs. Histotypes such as angiosarcoma, Ewing sarcoma, and high-grade UPS concentrate their metastatic events within the first year, making that window pivotal for frequent thoracic imaging and potentially timely systemic therapy [7,8,9,10,11,12,13,14]. By contrast, tumors that metastasize late—clear-cell sarcoma, low-grade fibromyxoid sarcoma, and myxoid liposarcoma—exhibit protracted risk and may benefit more from sustained, lower-frequency but prolonged monitoring, coupled with deferred systemic intervention.

Metastatic burden further nuances management [30,31,32,33]. Patients with a limited number of pulmonary nodules are plausible candidates for local therapy aimed at durable control, whereas those presenting with widespread multi-organ disease may require immediate systemic strategies [31,34,35]. Imaging modality choice should also align with tropism; thus, liver MRI or abdominal CT is warranted when hepatic spread dominates, skeletal MRI for bone-predominant histologies, and chest CT suffices for lung-only patterns. These reflections are hypotheses for prospective validation rather than being immediate directives.

Strengths of this study include prospective data capture, unified imaging pathways, and the central review, which minimized ascertainment bias. Limitations reflect the rarity of ultra-rare histotypes, potential referral bias, and a median follow-up of ~21 months, which may under-detect very late recurrences. Finally, while the study illuminates patterns, it was not designed to test whether tailored surveillance or burden-adapted treatment improve survival—an essential next step. Pandemic-related scheduling strain may have introduced a minor ascertainment lag; such delays would bias events toward later detection and thus render the observed early clustering as being conservative.

This work provides a descriptive template rather than a prescriptive algorithm. Three avenues merit prospective evaluation. First, pattern-stratified surveillance and treatment trials should test whether imaging intensity and therapeutic modality matched to timing, tropism, and metastatic burden translate into improved outcomes and enable biomarker-guided selection. Second, systematic appraisal of local ablative interventions is needed to ascertain which dissemination patterns derive the greatest survival or quality-of-life benefit. Finally, health-economic modelling must balance the cost of additional imaging and interventions against the potential gains from the timely, pattern-adapted management in high-risk subgroups.

## 5. Conclusions

A uniform, grade-based sarcoma follow-up paradigm is no longer defensible. Our real-world data reveal distinct timelines, destinations, and burdens of metastatic spread that can be harnessed to personalize surveillance and therapy. Adopting a histotype-specific, burden- and timing-adapted algorithm promises earlier detection for high-risk patients, reduced imaging exposure for low-risk groups, and the rational allocation of healthcare resources. Multicenter validation—and eventual guideline integration—are now imperative.

## Figures and Tables

**Table 1:** Baseline characteristics (age at diagnosis, sex) and the distribution of initial metastatic sites at presentation by histologic subtype (N = 93). Cells show n (% within histology). “Total initial metastasis sites” is the sum of sites observed across patients within a subtype; the multiplier in parentheses (e.g., 2.3×) denotes the mean number of metastatic sites per patient at diagnosis. Follow-up is reported as median [IQR] months from diagnosis to last contact or death (median follow-up may be estimated by reverse Kaplan–Meier).

Factor	Level	Overall	Non-Metastatic	Metastatic	*p*
n		295	202	93	
Sex (%)	Male	151 (51.2)	101 (50)	50 (53.8)	0.634
	female	144 (48.8)	101 (50)	43 (46.2)	
Age (median years (IQR)		58.1 (46.7; 71.2)	59.1 (47.7; 73.0)	56.4 (41.8; 67.8)	0.067
Follow-up (median months (IQR)		20.9 (9.9; 36.2)	18.9 (8.5; 33.4)	23.9 (14.2; 41.5)	0.025
Histologic Type (%)	Angiosarcoma of soft tissues	9 (3.1)	3 (1.5)	6 (6.5)	0.002
	Chondrosarcoma grade II, grade III	9 (3.1)	8 (4.0)	1 (1.1)	
	Dedifferentiated liposarcoma	28 (9.5)	24 (11.9)	4 (4.3)	
	Ewing sarcoma	9 (3.1)	3 (1.5)	6 (6.5)	
	Leiomyosarcoma (excluding skin)	31 (10.5)	17 (8.4)	14 (15.1)	
	Myxofibrosarcoma	22 (7.5)	20 (9.9)	2 (2.2)	
	Myxoid liposarcoma	19 (6.4)	15 (7.4)	4 (4.3)	
	Solitary fibrous tumor	13 (4.4)	10 (5.0)	3 (3.2)	
	Undifferentiated sarcoma (UPS)	53 (18.0)	35 (17.3)	18 (19.4)	
	Other	102 (34.6)	67 (33.29	35 (37.6)	
Grading (%)	Suspicious of malignancy	10 (3.4)	10 (5.0)	0 (0.0)	<0.001
	G1	62 (21.0)	55 (27.2)	7 (7.5)	
	G2	61 (20.7)	48 (23.8)	13 (14.0)	
	G2/3	6 (2.0)	3 (1.5)	3 (3.2)	
	G3	136 (46.1)	75 (37.1)	61 (65.6)	
	N/A	20 (6.8)	11 (5.4)	9 (9.7)	
Primary Tumor Size (median mm (IQR)		85.0 (51.8; 135.0)	80.0 (50.0; 130.0)	90.0 (63.0; 140.0)	0.175
Primary Tumor Site (%)	Extraperitoneal	37 (11.5)	25 (11.8)	12 (10.8)	0.090
	Head/Neck	15 (4.7)	10 (4.7)	5 (4.5)	
	Intraperitoneal	18 (5.6)	8 (3.8)	10 (9.0)	
	Intrathoracic	19 (5.9)	10 (4.7)	9 (8.1)	
	Lower limb	103 (32.0)	8 (3.8)	31 (27.9)	
	Lower limb girdle	46 (14.3)	29 (13.7)	17 (15.3)	
	Trunk wall	42 (13.0)	33 (15.6)	9 (8.1)	
	Upper limb	22 (6.8)	14 (6.6)	8 (7.2)	
	Upper limb girdle	8 (2.5)	6 (2.8)	2 (1.8)	
	Viscera	12 (3.7)	4 (1.9)	8 (7.2)	

**Table 2:** (**a**) Initial Metastasis by histologic subtype. Distribution of initial metastatic sites at diagnosis by histologic subtype (N = 93). Each cell shows number of patients with ≥1 lesion at the indicated site followed by the percent of patients within that histologic subtype in parentheses. “Total Initial Metastases Sites” gives the sum of sites observed across patients within each subtype; the value in parentheses (e.g., 2.3×) denotes the mean number of metastatic sites per patient in that subtype at diagnosis. Patients can contribute to multiple sites; therefore row sums exceed the number of patients (**b**) Any-time metastases by histologic subtype. Distribution of metastatic sites at any time during the disease course by histologic subtype (N = 93). Cells report count of patients and % within subtype. “Total Metastasis Sites” represents the cumulative number of sites recorded per subtype over the course of follow-up; the parenthetical multiplier (e.g., 3.2×) indicates the mean number of metastatic sites per patient in that subtype across time. Patients may appear in multiple sites.

**(a)**														
**Histologic Type**	**Bone**	**CNS/Meninges**	**Head/Neck**	**Intraperitoneal**	**Liver**	**Lungs**	**Lymph Nodes**	**Retroperitoneal**	**Skeletal Muscle**	**Skin/Subcutaneous Tissue**	**Thorax**	**Other**	**Total Initial Metastases Sites**	**Total Patients**
Angiosarcoma	3 (42.9)	0 (0.0)	0 (0.0)	2 (28.6)	1 (14.3)	2 (28.6)	4 (57.1)	0 (0.0)	2 (28.6)	1 (14.3)	1 (14.3)	0 (0.0)	16 (2.3×)	7
Chondrosarcoma	2 (28.6)	0 (0.0)	0 (0.0)	0 (0.0)	0 (0.0)	5 (71.4)	1 (14.3)	0 (0.0)	0 (0.0)	0 (0.0)	0 (0.0)	0 (0.0)	8 (1.1×)	7
Epithelioid hemangioendothelioma	2 (66.7)	0 (0.0)	0 (0.0)	0 (0.0)	2 (66.7)	2 (66.7)	0 (0.0)	0 (0.0)	1 (33.3)	1 (33.3)	0 (0.0)	0 (0.0)	8 (2.7×)	3
Ewing sarcoma	5 (83.3)	0 (0.0)	0 (0.0)	0 (0.0)	0 (0.0)	3 (50.0)	2 (33.3)	0 (0.0)	1 (16.7)	0 (0.0)	0 (0.0)	1 (16.7)	12 (2.0×)	6
Leiomyosarcoma (excluding skin)	1 (7.1)	0 (0.0)	0 (0.0)	1 (7.1)	1 (7.1)	11 (78.6)	1 (7.1)	0 (0.0)	3 (21.4)	0 (0.0)	0 (0.0)	0 (0.0)	18 (1.3×)	14
Liposarcoma (dediff./myx./pleom.)	0 (0.0)	1 (10.0)	0 (0.0)	0 (0.0)	0 (0.0)	5 (50.0)	1 (10.0)	1 (10.0)	1 (10.0)	1 (10.0)	0 (0.0)	2 (20.0)	12 (1.2×)	10
Osteosarcoma	1 (25.0)	0 (0.0)	0 (0.0)	0 (0.0)	0 (0.0)	3 (75.0)	0 (0.0)	0 (0.0)	0 (0.0)	0 (0.0)	0 (0.0)	0 (0.0)	4 (1.0×)	4
Pleomorphic dermal sarcoma	1 (25.0)	0 (0.0)	0 (0.0)	0 (0.0)	0 (0.0)	1 (25.0)	1 (25.0)	0 (0.0)	0 (0.0)	1 (25.0)	0 (0.0)	0 (0.0)	4 (1.0×)	4
Solitary fibrous tumor	1 (33.3)	0 (0.0)	0 (0.0)	0 (0.0)	1 (33.3)	2 (66.7)	0 (0.0)	0 (0.0)	0 (0.0)	0 (0.0)	0 (0.0)	0 (0.0)	4 (1.3×)	3
Undifferentiated sarcoma (UPS)	0 (0.0)	0 (0.0)	0 (0.0)	1 (5.6)	1 (5.6)	13 (72.2)	2 (11.1)	0 (0.0)	0 (0.0)	0 (0.0)	0 (0.0)	1 (5.6)	18 (1.0×)	18
Other	1 (5.9)	1 (5.9)	1 (5.9)	3 (17.6)	5 (29.4)	11 (64.7)	2 (11.8)	3 (17.6)	1 (5.9)	1 (5.9)	2 (11.8)	1 (5.9)	32 (1.9×)	17
Total	17 (18.3)	2 (2.2)	1 (1.1)	7 (7.5)	11 (11.8)	58 (62.4)	14 (15.1)	4 (4.3)	9 (9.7)	5 (5.4)	3 (3.2)	5 (5.4)	136 (1.5×)	93
**(b)**														
**Histologic Type**	**Bone**	**CNS/Meninges**	**Head/Neck**	**Intraperitoneal**	**Liver**	**Lungs**	**Lymph Nodes**	**Retroperitoneal**	**Skeletal Muscle**	**Skin/Subcutaneous Tissue**	**Thorax**	**Other**	**Total Metastasis Sites**	
Angiosarcoma	4 (57.1)	1 (14.3)	1 (14.3)	2 (28.6)	2 (28.6)	4 (57.1)	5 (71.4)	0 (0.0)	3 (42.9)	1 (14.3)	1 (14.3)	0 (0.0)	24 (3.4×)	
Chondrosarcoma	4 (57.1)	1 (14.3)	1 (14.3)	1 (14.3)	0 (0.0)	7 (100.0)	3 (42.9)	0 (0.0)	0 (0.0)	0 (0.0)	1 (14.3)	1 (14.3)	19 (2.7×)	
Epithelioid hemangioendothelioma	3 (100.0)	0 (0.0)	0 (0.0)	0 (0.0)	2 (66.7)	3 (100.0)	0 (0.0)	0 (0.0)	1 (33.3)	3 (100.0)	0 (0.0)	0 (0.0)	12 (4.0×)	
Ewing sarcoma	6 (100.0)	1 (16.7)	1 (16.7)	0 (0.0)	1 (16.7)	6 (100.0)	2 (33.3)	1 (16.7)	1 (16.7)	1 (16.7)	0 (0.0)	1 (16.7)	21 (3.5×)	
Leiomyosarcoma (excluding skin)	6 (42.9)	1 (7.1)	0 (0.0)	2 (14.3)	3 (21.4)	14 (100.0)	4 (28.6)	2 (14.3)	5 (35.7)	2 (14.3)	0 (0.0)	2 (14.3)	41 (2.9×)	
Liposarcoma (dediff./myx./pleom.)	3 (30.0)	2 (20.0)	0 (0.0)	1 (10.0)	1 (10.0)	8 (80.0)	2 (20.0)	3 (30.0)	4 (40.0)	3 (30.0)	3 (30.0)	2 (20.0)	32 (3.2×)	
Osteosarcoma	2 (50.0)	0 (0.0)	1 (25.0)	1 (25.0)	0 (0.0)	3 (75.0)	1 (25.0)	1 (25.0)	1 (25.0)	0 (0.0)	1 (25.0)	0 (0.0)	11 (2.8×)	
Pleomorphic dermal sarcoma	2 (50.0)	1 (25.0)	0 (0.0)	0 (0.0)	0 (0.0)	2 (50.0)	2 (50.0)	1 (25.0)	2 (50.0)	0 (0.0)	0 (0.0)	0 (0.0)	11 (2.8×)	
Solitary fibrous tumor	2 (66.7)	0 (0.0)	0 (0.0)	1 (33.3)	2 (66.7)	2 (66.7)	1 (33.3)	0 (0.0)	0 (0.0)	0 (0.0)	1 (33.3)	0 (0.0)	9 (3.0×)	
Undifferentiated sarcoma (UPS)	1 (5.6)	1 (5.6)	0 (0.0)	2 (11.1)	1 (5.6)	14 (77.8)	4 (22.2)	2 (11.1)	3 (16.7)	2 (11.1)	2 (11.1)	1 (5.6)	33 (1.8×)	
Other	4 (23.5)	1 (5.9)	1 (5.9)	5 (29.4)	6 (35.3)	15 (88.2)	6 (35.3)	5 (29.4)	2 (11.8)	1 (5.9)	6 (35.3)	1 (5.9)	53 (3.1×)	
Total	37 (39.8)	9 (9.7)	5 (5.4)	15 (16.1)	18 (19.4)	78 (83.9)	30 (32.3)	15 (16.1)	21 (22.6)	15 (16.1)	15 (16.1)	8 (8.6)	266 (2.9×)	

**Table 3:** Metastasis site × histology with prevalence. Cross-tabulation of metastatic site by histologic subtype with cohort totals and prevalence. Cells contain the number of patients with ≥1 lesion at the site and the % of patients within that subtype. Bottom rows summarize: Total Metastasis Sites (sum across sites per subtype, with mean sites per patient in parentheses), Total Patients, counts with/without metastasis, and Prevalence of Metastasis (patients with any metastasis ÷ total patients per subtype). Patients may contribute to multiple sites.

Metastasis Site	Angiosarcoma	Chondrosarcoma	Epithelioid Hemangioendothelioma	Ewing Sarcoma	Leiomyosarcoma (Excluding Skin)	Liposarcoma (dediff./myx./pleom./NOS)	Osteosarcoma	Pleomorphic Dermal Sarcoma	Solitary Fibrous Tumor	Undifferentiated Sarcoma (UPS)	Other	Total
Bone	4 (57.1)	4 (57.1)	3 (100.0)	6 (100.0)	6 (42.9)	3 (30.0)	2 (50.0)	2 (66.7)	1 (5.6)	4 (23.5)	37 (39.8)	
CNS/Meninges	1 (14.3)	1 (14.3)	0 (0.0)	1 (16.7)	1 (7.1)	2 (20.0)	0 (0.0)	1 (25.0)	0 (0.0)	1 (5.6)	1 (5.9)	9 (9.7)
Head/Neck	1 (14.3)	1 (14.3)	0 (0.0)	1 (16.7)	0 (0.0)	0 (0.0)	1 (25.0)	0 (0.0)	0 (0.0)	0 (0.0)	1 (5.9)	5 (5.4)
Intraperitoneal	2 (28.6)	1 (14.3)	0 (0.0)	0 (0.0)	2 (14.3)	1 (10.0)	1 (25.0)	0 (0.0)	1 (33.3)	2 (11.1)	5 (29.4)	15 (16.1)
Liver	2 (28.6)	0 (0.0)	2 (66.7)	1 (16.7)	3 (21.4)	1 (10.0)	0 (0.0)	0 (0.0)	2 (66.7)	1 (5.6)	6 (35.3)	18 (19.4)
Lungs	4 (57.1)	7 (100.0)	3 (100.0)	5 (100.0)	14 (100.0)	8 (80.0)	3 (75.0)	2 (50.0)	2 (66.7)	14 (77.8)	15 (88.2)	78 (83.9)
Lymph nodes	5 (71.4)	3 (42.9)	0 (0.0)	2 (33.3)	4 (28.6)	2 (20.0)	1 (25.0)	2 (50.0)	1 (33.3)	4 (22.2)	6 (35.3)	30 (32.3)
Skeletal muscle	3 (42.9)	0 (0.0)	1 (33.3)	1 (16.7)	5 (35.7)	4 (40.0)	1 (25.0)	1 (25.0)	0 (0.0)	3 (16.7)	2 (11.8)	21 (22.6)
Skin/Subcutaneous tissue	1 (14.3)	0 (0.0)	3 (100.0)	1 (16.7)	2 (14.3)	3 (30.0)	0 (0.0)	0 (0.0)	1 (33.3)	2 (11.1)	1 (5.9)	15 (16.1)
Thorax	1 (14.3)	1 (14.3)	0 (0.0)	0 (0.0)	3 (30.0)	1 (25.0)	0 (0.0)	1 (33.3)	2 (11.1)	0 (0.0)	6 (35.3)	15 (16.1)
Retroperitoneal	0 (0.0)	0 (0.0)	0 (0.0)	1 (16.7)	2 (14.3)	3 (30.0)	1 (25.0)	1 (25.0)	0 (0.0)	2 (11.1)	5 (29.4)	15 (16.1)
Other	0 (0.0)	1 (14.3)	0 (0.0)	1 (16.7)	2 (14.3)	2 (20.0)	0 (0.0)	0 (0.0)	0 (0.0)	1 (5.6)	1 (5.9)	8 (8.6)
Total Metastasis Sites	24 (3.4×)	19 (2.7×)	12 (4.0×)	21 (3.5×)	41 (2.9×)	32 (3.2×)	11 (2.8×)	11 (2.8×)	9 (3.0×)	33 (1.8×)	53 (3.1×)	266 (2.9×)
Total Patients	10	18	5	9	31	52	7	6	13	53	91	295
…without Metastasis	3	11	2	3	17	42	3	2	10	35	74	202
…with Metastasis	7	7	3	6	14	10	4	4	3	18	17	93
Prevalence of Metastasis	70.0%	38.9%	60.0%	66.7%	45.2%	19.2%	57.1%	66.7%	23.1%	34.0%	18.7%	31.5%

## Data Availability

The data presented in this study are available on request from the corresponding author.
